# Measuring the Adsorption of Electrolytes on Lipid Monolayers

**DOI:** 10.1021/acs.jpclett.3c00795

**Published:** 2023-05-11

**Authors:** Boyan Peychev, Dimitrinka Arabadzhieva, Ivan Minkov, Elena Mileva, Stoyan K. Smoukov, Radomir I. Slavchov

**Affiliations:** † Queen Mary University of London, School of Engineering and Materials Science, Mile End Road, London E1 4NS, United Kingdom; ‡ Rostislaw Kaischew Institute of Physical Chemistry, Bulgarian Academy of Sciences, Acad. G. Bonchev Str., bl. 11, 1113 Sofia, Bulgaria; ¶ Department of Chemistry, Biochemistry, Physiology, and Pathophysiology, Faculty of Medicine, Sofia University, 1 Koziak Str., 1407 Sofia, Bulgaria

## Abstract

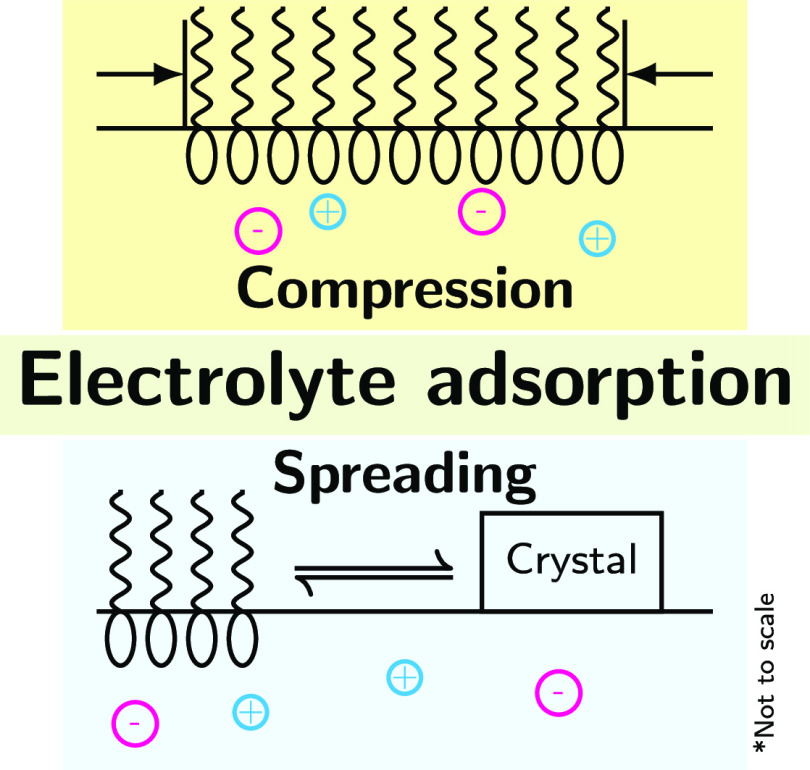

The interactions between ions and lipid monolayers have captivated the attention of biologists and chemists alike for almost a century. In the absence of experimentally accessible concentration profiles, the electrolyte adsorption remains the most informative quantitative characteristic of the ion-lipid interactions. However, there is no established procedure to obtain the electrolyte adsorption on spread lipid monolayers. As a result, in the literature, the ion-lipid monolayer interactions are discussed qualitatively, based on the electrolyte effect on more easily accessible variables, e.g., surface tension. In this letter, we demonstrate how the electrolyte adsorption on lipid monolayers can be obtained experimentally. The procedure requires combining surface pressure versus molecular area compression isotherms with spreading pressure data. For the first time, we report an adsorption isotherm of NaCl on a lipid monolayer as a function of the density of the monolayer. The leading interactions seem to be the osmotic effect from the lipid head groups in the surface layer and ion-lipid association.

It is well-established that ions play an important role in many membrane processes, such as regulating the membrane surface potential,^1^ transmembrane transport,^1−3^ and signal transduction,^4^ etc. Inorganic electrolytes have been shown to affect the physicochemical properties of lipid structures,^5^ for instance, they change the melting temperature,^1,6−11^ the headgroup tilt,^12,13^ and the morphology^1,14,15^ of mono- and bilayers. Not surprisingly, these effects are ion specific—dependent on the chemical identity of the constituent ions as well as their concentration. Because of the importance of membrane phenomena, the ion-lipid interactions have been a subject of intensive study via a multitude of experimental techniques, e.g., differential scanning calorimetry,^6,9,10^ X-ray diffraction,^10^ electron paramagnetic resonance,^8,16^ nuclear magnetic resonance,^12^ Brewster angle microscopy,^15,17^ grazing incidence X-ray diffraction,^17^ infrared reflection–absorption spectroscopy,^15,17,18^ sum frequency generation,^14^ and chemical trapping.^19^ Of course, we cannot omit the classical equation of state studies (surface pressure π vs area *S*) of lipid monolayers on aqueous electrolyte solutions done in a Langmuir trough.^15,17,20^ Remarkably, despite decades of effort, perhaps the most direct macroscopic characteristic of the ion-lipid interactions—the electrolyte adsorption Γ_el_—remains elusive. That is due to the fact that, for a three component system, Γ_el_ cannot be extracted from a single monolayer compression isotherm alone. In this letter, it is our aim to demonstrate a new thermodynamic method to measure electrolyte adsorption on monolayers and apply it to a lipid system. The method is based on the work of Frumkin and Pankratov from 1939,^21^ but has been realized only recently.^22^ The idea of the method is to combine compression isotherms data with equilibrium spreading pressure measurements for the amphiphile used as a reference state of fixed chemical potential. The method can be simple from an experimental point of view, but involves a somewhat intricate computational procedure. Here, we present the procedure for data handling in a simplified, comprehensible way, and apply it for the first time ever to the system NaCl/dipalmitoylphosphatidylcholine (DPPC) monolayer.

In a two component solute/solvent system, to determine the adsorption of the solute, it is sufficient to measure the surface tension σ as a function of concentration *C*_el_, and then use the Gibbs isotherm.^23,24^ However, this method is not applicable when a third component—the amphiphile monolayer—is added. The Gibbs isotherm for a lipid monolayer spread on an electrolyte solution reads
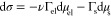
1where ν is the isotonic coefficient of the electrolyte, Γ_s_ = 1/*S* is the surface concentration of lipid molecules (monolayer density), μ_s_ is the chemical potential of the lipid surfactant, and μ_el_ is the bulk chemical potential of the electrolyte. The chemical potential of the electrolyte follows from its concentration; μ_el_ ≡ μ_el_^°^ + *RT* ln γ_el_*C*_el_, where γ_el_ and *C*_el_ are the electrolyte activity coefficient and concentration. The electrolyte adsorption that follows from eq 1 is
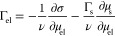
2When compression isotherms σ(Γ_s_, *C*_el_) of lipid monolayers are measured on a substrate with concentration *C*_el_, both Γ_el_ and μ_s_ are functions of the independent variables Γ_s_ and *C*_el_. Thus, while for monolayer-free surfaces (Γ_s_ = 0), the surface tension data fixes Γ_el_ through the first term in eq 2, this is not the case when a lipid is adsorbed — in this case, the effect of the electrolyte on μ_s_ remains undetermined. The σ(Γ_s_, *C*_el_) isotherms simply do not encode sufficient information to extract the electrolyte adsorption. For this reason, in the literature, the tensionmetric results for the effect of the electrolyte on lipid monolayers are discussed qualitatively, in terms of the effect of the electrolyte on the ’cohesion’ of the monolayer based on the change of molecular area intercept^25^ or on the vertical/horizontal shift of the isotherm.^15,17,18,18^ The actual electrolyte adsorption Γ_el_ and its variation with the monolayer density Γ_s_ remain unknown for even the simplest phospholipids.

From eq 1, the following partial differential relations can be derived:
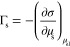
3
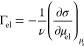
4and
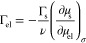
5From eq 3, it can be seen that a compression isotherm, at one fixed electrolyte concentration, defines the change of the surfactant chemical potential μ_s_(σ) as
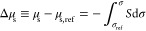
6where μ_s,ref_ is an integration constant corresponding to a chosen reference state {*S*_ref_, σ_ref_} of the monolayer.^21,22^ Once μ_s_(Γ_s_, *C*_el_) is known, eq 4 and 5 provide two ways to calculate the electrolyte adsorption from compression isotherms at several electrolyte concentrations. The missing piece of information is the dependence of the integration constant μ_s,ref_ in eq 6, intrinsic to the calculation of μ_s_, on *C*_el_. Fortunately, due to the nature of eqs 4 and 5, as long as the reference state is chosen such that μ_s,ref_ is independent of *C*_el_, the value of this integration constant becomes irrelevant.

In general, when a crystal or droplet of insoluble surfactant is put in contact with the aqueous surface, the surfactant molecules spread on the surface to produce a dense monolayer. Such a monolayer at equilibrium with the bulk surfactant phase is known as an *equilibrium spread monolayer*. The key idea of Frumkin and Pankratov that allows Γ_el_ to be extracted was to use the equilibrium spread monolayer as a reference state {*S*_sp_, σ_sp_} in eq 6.^21^ In that case, the integration constant μ_s,ref_ = μ_s,sp_ is the chemical potential of the amphiphile in the bulk phase. Arguably, the electrolyte cannot penetrate into the bulk surfactant phase and, therefore, the chemical potential μ_s,sp_ of the equilibrium spread monolayer is electrolyte independent. The bulk surfactant phase acts as a chemical potential reservoir. Frumkin and Pankratov laid the groundwork for the method by combining data for compression isotherms and equilibrium spreading tension and comparing the change of the surface pressure at constant chemical potential (i.e., eq 4) of ethyl palmitate monolayer on aqueous KI. Only recently, we resurrected their approach to determine quantitatively the electrolyte excess on several nonionic surfactant monolayers^22,26^ and extended it by using the other route of calculating Γ_el_, via eq 5. However, the method has not yet been applied to phospholipid monolayers.

Data for compression isotherms of various combinations of lipid monolayer and electrolytes have been reported in the literature.^15,17^ In this letter, we use the compression isotherms data by Adams et al.^15^ for DPPC (dipalmitoylphosphatidylcholine) on NaCl solutions to calculate the electrolyte adsorption onto the monolayer. In order to do that, their data must be combined with measurements of the equilibrium spreading pressure π_sp_ of crystals of DPPC on aqueous solutions of NaCl. We measured the spreading pressure π_sp_ = σ_0_ – σ_sp_ of DPPC crystals to obtain π_sp_ = 44.1, 46.5, and 47.0 ± 0.3 mN/m at *C*_el_ = 0, 0.6, and 2.0 M NaCl, respectively (see Figure B.1 in the SI; σ_0_ is the surface tension of the respective NaCl solutions).

As a first step in the calculation procedure, eq 6 is used to determine the chemical potential change Δμ_s_ of DPPC (with respect to the DPPC crystalline phase) at the three NaCl concentrations. The detail description of this step is given in the SI. The results are presented in Figure 1 and already provide insight into the system. As seen, at a constant surface pressure, the chemical potential of DPPC decreases as *C*_el_ increases, i.e., the electrolyte stabilizes the monolayer indicating attractive ion-monolayer interactions. This is not at all obvious from the compression isotherms (see Figure C.1 in the SI)—at a constant π the addition of electrolyte leads to expansion of the lipid monolayer, which can be erroneously interpreted as destabilization, i.e., increase of the lateral lipid–lipid repulsion.

**Figure 1:**
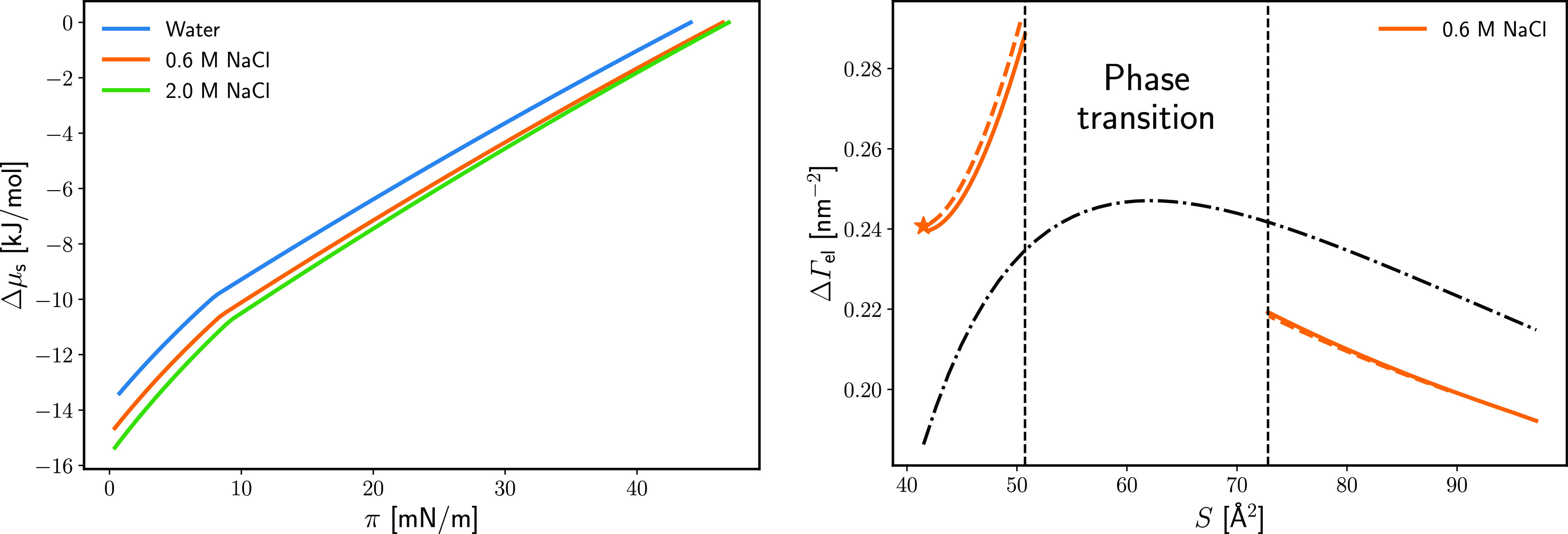
(left) The change of the chemical potential Δμ_s_ of DPPC monolayer as a function of the surface pressure at different concentrations of NaCl in the subphase (see eq 6). (right) Monolayer-induced adsorption of electrolyte ΔΓ_el_ as a function of the DPPC area per molecule *S* at 0.6 M NaCl. The solid lines are calculated at constant surfactant chemical potential μ_s_. The dashed lines are calculated at a constant surface pressure π. The star is calculated straight from the spreading pressure data (see SI-D). The dash-dotted line shows a simple electrolyte adsorption model based on complexation and excluded volume interactions (see SI-E).

With Δμ_s_ known, as a second step, the electrolyte adsorption is calculated via numerical differentiation, either through eq 4 (Frumkin’s approach) or eq 5(22) (see details in SI section D). It is convenient to express the results as *monolayer-induced adsorption*, , i.e., the electrolyte excess attracted to the surface by the monolayer, compared to the monolayer-free surface. ΔΓ_el_ is a derivative of the surface pressure π rather than of σ (see eqs D.2 and D.3 in the SI). The numerical differentiation with respect to *C*_el_ is far more accurate for the central point (0.6 M NaCl) than for the terminal points 0 and 2 M. The calculated dependence of ΔΓ_el_ on the lipid area per molecule *S* is presented in Figure 1 for 0.6 M NaCl. The two approaches for determining ΔΓ_el_ (via eqs 4 and 5) lead to almost identical ΔΓ_el_ values, which is a test of the thermodynamic compatibility of the two sets of data—compression isotherm and equilibrium spreading pressure. It can be seen that the electrolyte adsorption on the lipid monolayer is more positive than that on water|air, i.e., the monolayer attracts NaCl. Furthermore, the compression of the monolayer leads to an increase of ΔΓ_el_ up to the phase transition point of the monolayer. However, in the condensed monolayer region, ΔΓ_el_ starts to decrease, i.e., the electrolyte adsorption is maximum at a certain intermediate density of the monolayer. This is a common behavior also found for simple electrolytes on alcohol,^26^ carboxylic acid, and ester monolayers^22^ and implies a complex interaction landscape between ions and monolayers.

The decrease in electrolyte adsorption at high density of the monolayer is in line with the “squeezing out” effect discussed by Aroti et al.^17,18^ The initial increase in Γ_el_ with Γ_s_ is largely due to the osmotic effect caused by the polar headgroups of the amphiphile that effectively dilute the water in the surface layer.^22,26^ However, to explain the complicated nonmonotonous relationship between Γ_el_ and Γ_s_, specific interactions between the lipid and the ions must be present as well, e.g., ion-headgroup complexation,^22,27^ ion-surface dipole interaction,^28^ etc. In the Supporting Information we propose a simple electrolyte adsorption isotherm (see SI-E). This model agrees with the experimental data within 10% in the liquid expanded region and within 20% in the condensed, see Figure 1-right.

In summary, we demonstrated that the adsorption of electrolytes on lipid monolayers can be determined experimentally using the method of Frumkin-Pankratov^22^ on the example of NaCl on DPPC. The obtained NaCl adsorption vs DPPC monolayer density is, to our knowledge, the first reported of its kind, and shows a behavior similar to that observed previously for simpler surfactants.^22,26^ In comparison to simple ions interacting with monolayer-free interfaces,^23,24^ the system studied here already highlights the presence of complex ion-lipid interaction, and it does so in a more direct quantitative manner than other experimental approaches.

The Frumkin-Pankratov method is inexpensive from an experimental point of view, but involves a relatively complex computation procedure (developed in detail previously^22^ and presented here at an algorithmic level in the SI, for ease of reference). However, the information that the Frumkin-Pankratov method provides is an important addition to the fundamental understanding of the role of the electrolytes in the structure of the lipid membranes. The data for electrolyte adsorption on monolayers can also be very useful for validation of theoretical models^29,30^ and molecular dynamics simulations.^27,31,32^

## Methods

DPPC crystals (>99%) from Avanti Polar lipids and NaCl (99.8%) from Sigma-Aldrich were used. The NaCl was calcinated at 400 °C before use to remove any surface active impurities. The surface tension was measured with a platinum Wilhelmy plate attached to a KSV Nima surface balance. The solution temperature was kept constant at 25 ± 1 °C with a Lauda Eco Silver RE415 thermostat. For the calculations in the current study we used the compression isotherm data of Adams et al.^15^ The spreading pressure of the lipid was determined using the standard procedure,^33,34^ modified by adding an organic solvent to facilitate the spreading process. In a typical experiment, approximately 10 ± 1 mg of DPPC crystals are deposited on the cleaned surface of the electrolyte solution. Unlike lower molecular weight surfactants, the DPPC crystals do not spread readily over the surface. In order to facilitate the formation of a monolayer in contact with DPPC crystals, 30–50 μL of chloroform is added with a Hamilton syringe in a dropwise manner. The results are distributed around an average, which is assumed to be the spreading equilibrium, with a standard error ∼0.3 mN/m (see Figure D.1 in the SI).

## Supporting Information Available

A general algorithm for calculating monolayer-induced adsorption of electrolytes on lipid monolayers; a simple model for electrolyte adsorption on lipid monolayers (PDF)
