# Supplementary material for: Measuring the Adsorption of Electrolytes on Lipid Monolayers
Source: J Phys Chem Lett. 2023 May 11;14(20):4652–6. doi: 10.1021/acs.jpclett.3c00795 (PMC10226113; doi:10.1021/acs.jpclett.3c00795)
Supplement: Supplementary file 1 — jz3c00795_si_001.pdf [file jz3c00795_si_001.pdf]

Supplementary Information: Measuring the Adsorption of Electrolytes on Lipid Monolayers

Boyan Peychev[a], Dimitrinka Arabadzhieva[b], Ivan Minkov[c,b], Elena Mileva[b], Stoyan K. Smoukov[a], and Radomir I. Slavchov[a,*]

[a] *Queen Mary University of London, School of Engineering and Materials Science, Mile End Road, London E1 4NS, United Kingdom*

[b] *Rostislaw Kaischew Institute of Physical Chemistry, Bulgarian Academy of Sciences, Acad. G. Bonchev Str., bl. 11, 1113 Sofia, Bulgaria*

[c] *Department of Chemistry, Biochemistry, Physiology, and Pathophysiology, Faculty of Medicine, Sofia University, 1 Koziak Str., 1407 Sofia, Bulgaria*

[*] *r.slavchov@qmul.ac.uk*

A Solution properties

- The molal concentration $C_{\mathrm{el,m}}$ is calculated as

$$C_{\mathrm{el,m}} = \frac{C_{\mathrm{el}}}{\rho_{\mathrm{w}}(1 - V_{\mathrm{el}}C_{\mathrm{el}} - S_{\rho}C_{\mathrm{el}}^{3/2})}, \tag{A.1}$$

where C_{el} is the molar concentrations, V_{el} is the partial molar volume of the aqueous electrolyte, and S_ρ is an empirical correction factor.

- The molal-based activity coefficient $\gamma_{\mathrm{el,m}}$ is calculated as

$$\lg \gamma_{\mathrm{el,m}} = -\frac{A\sqrt{C_{\mathrm{el,m}}}}{1+B\sqrt{C_{\mathrm{el,m}}}} + \sum_{i=1}^{i\leq 6} \beta_i C_{\mathrm{el,m}}^i, \tag{A.2}$$

where A and B are Debye-Hückel constants and β_i are empirical polynomial coefficients.

- The osmotic pressure p_{osm} of the electrolyte solution is calculated as

$$p_{\mathrm{osm}} = RT\rho_{\mathrm{w}} \int_0^{C_{\mathrm{el,m}}} C_{\mathrm{el,m}} \frac{\mathrm{d}\ln \gamma_{\mathrm{el,m}} C_{\mathrm{el,m}}}{\mathrm{d}C_{\mathrm{el,m}}} \mathrm{d}C_{\mathrm{el,m}}, \tag{A.3}$$

where R is the universal gas constant, T is the temperature, and ρ_{w} is the density of water.

Table A.1: The parameters used for the calculations

**Constants**					
R [J/K mol]	ρ_{w} [kg/m^3]	A [(mol/kg)$^{1/2}$]			
8.314	997.0	0.51			
**NaCl constants**					
V_{el} [mL/mol]	S_ρ [(L/mol)$^{3/2}$]	B [(mol/kg)$^{1/2}$]	β_1 [(mol/kg)]	β_2 [(mol/kg)^2]	β_3 [(mol/kg)^3]
16.4	2.153e-3	1.4495	2.0442e-2	5.793e-3	-2.89e-4

B Spreading pressure

Fig. B.1 shows the measured spreading pressure of DPPC on NaCl. The results are at a constant chemical potential of the surfactant. The direct numerical differentiation according to eq. D.4 allows the adsorption of electrolyte on the equilibrium spread monolayer to be calculated.

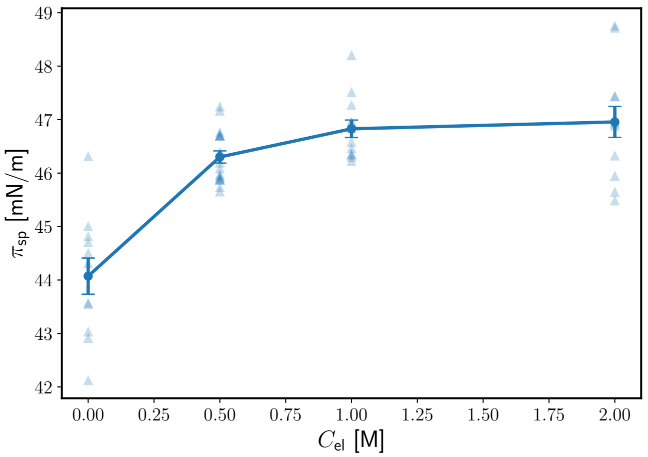

Figure B.1: The spreading pressure π_{sp} of DPPC on electrolytic solutions as a function of the electrolyte concentration C_{el} at 25 °C.

C Isotherms

- Working under the assumption that the dynamic isotherms are close enough to equilibrium, a spline function is used to calculate the molecular area S_{sp} at spreading pressure π_{sp} for each isotherm. The points above π_{sp} are non-equilibrium and are not considered.
- The point $\{S_{sp}, \pi_{sp}\}$ is added to the $\{S, \pi\}$ data set.
- Some of the highest molecular area points may correspond to the 2D gas-LE coexistence region and are not considered.
- The monolayer compressibility is calculated as

$$\kappa = -\frac{1}{S}\frac{\partial S}{\partial \pi}. \tag{C.1}$$

- The first-order phase transition from liquid to condensed monolayer should be a horizontal line (constant π) connecting the two single-phase regions. The non-linear behaviour observed around the phase transition plateau is assumed to be a kinetic effect due to the compression of a heterogeneous monolayer.[2,3] The surface pressure π_{pt}, that corresponds to the phase transition is determined as the value corresponding to maximum monolayer compressibility $\max(\kappa)$.

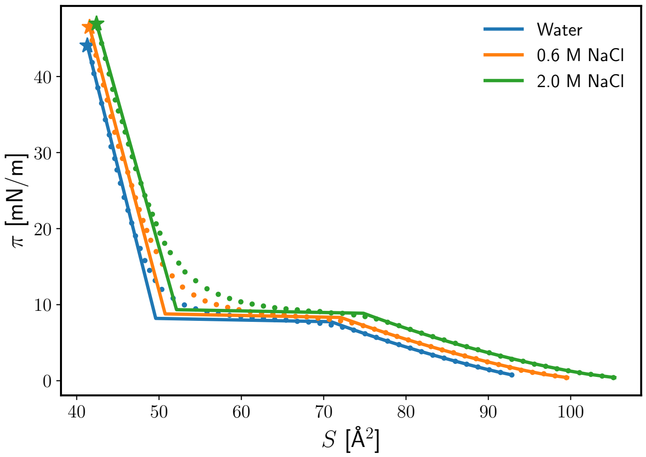

Figure C.1: The surface pressure π of DPPC monolayers as a function of the area per molecule S at different electrolyte concentrations C_{el}. Points are experimental data taken from Adams *et al.*.[1] Stars are the spreading pressures from figure B.1. Lines are a fit of the data (described in the text).

- The experimental points are divided in two subsets; C for the condensed phase ($\pi > \pi_{\mathrm{pt}} + 1$ mN/m) and LE for the liquid-expanded phase ($\pi < \pi_{\mathrm{pt}} - 1$ mN/m).

- The LE phase data are fitted (4 parametric unweighted regression) to a polylog function

$$S^{\mathrm{LE}} = S_{\mathrm{pt}} - c_1 \ln\left(\frac{\pi}{\pi_{\mathrm{pt}}}\right) - c_2 \ln^2\left(\frac{\pi}{\pi_{\mathrm{pt}}}\right) - c_3 \ln^3\left(\frac{\pi}{\pi_{\mathrm{pt}}}\right). \tag{C.2}$$

- In the condensed phase the surface pressure π is assumed to be a linear function of the molecular area S.

$$S^{\mathrm{C}} = S_{\mathrm{sp}} + c_4\left(1 - \frac{\pi}{\pi_{\mathrm{sp}}}\right) \tag{C.3}$$

  The data points are fitted (1 parametric weighted fit) to equation C.3 with weights π^2. Thus, the points closer to π_{sp} contribute more to the fit. The reason is that the data for the C region contain points for a heterogeneous monolayer – the LE 2D films between the C domains remain stable even at rather high surface pressure. Thus, the C points are less reliable the closer they are to π_{pt}.

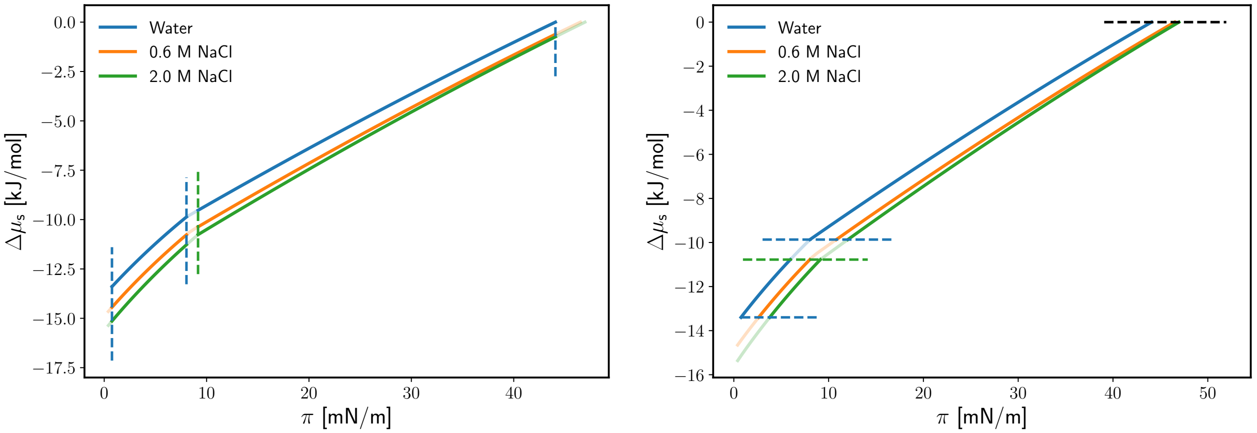

(a) Constant π (b) Constant $\Delta\mu_\mathrm{s}$

Figure D.1: The change of DPPC monolayer chemical potential $\Delta\mu_\mathrm{s}$ as a function of the surface pressure π at different electrolyte concentrations C_el. (a) Shows the differentiable range at constant surface pressure π (for equation D.2). (b) Shows the differentiable range at constant chemical potential μ (for equation D.3).

D Monolayer induced adsorption of electrolyte

- For each isotherm, the monolayer surface concentration is calculated as $\varGamma_\mathrm{s} = 1/S$.

- For each isotherm the chemical potential change is calculated as

$$\Delta\mu_\mathrm{s} = \begin{cases} c_4\left(\pi - \dfrac{\pi_\mathrm{sp}}{2} - \dfrac{\pi^2}{2\pi_\mathrm{sp}}\right) & \pi \geq \pi_\mathrm{pt} \\ \\ c_4\left(\pi - \dfrac{\pi_\mathrm{sp}}{2} - \dfrac{\pi^2}{2\pi_\mathrm{sp}}\right) + (2c_2 - c_1 - 6c_3)\ln\left(\dfrac{\pi}{\pi_\mathrm{pt}}\right)\pi & \\ -\,(c_2 - 3c_3)\ln^2\left(\dfrac{\pi}{\pi_\mathrm{pt}}\right)\pi - c_3\ln^3\left(\dfrac{\pi}{\pi_\mathrm{pt}}\right)\pi & \pi < \pi_\mathrm{pt} \\ +\,(S_\mathrm{pt} + c_1 - 2c_2 + 6c_3)(\pi - \pi_\mathrm{pt}) + S_\mathrm{sp}(\pi - \pi_\mathrm{sp}) & \end{cases} \tag{D.1}$$

- The minimum spreading pressure $\pi_\mathrm{sp,min}$ from all isotherms is determined.

- The minimum $\pi_\mathrm{pt,min}$ and maximum $\pi_\mathrm{pt,max}$ phase transition surface pressures are determined from all isotherms.

- The minimum $\Delta\mu_\mathrm{pt,min}$ and maximum $\Delta\mu_\mathrm{pt,max}$ phase transition chemical potential changes are determined from all isotherms.

- The highest minimal surface pressure $\pi_{\mathrm{min,max}}$ (corresponding to the highest molecular area in the data set) is determined from all isotherms.

- The highest minimal chemical potential change $\Delta\mu_{\mathrm{min,max}}$ (corresponding to the highest molecular area in the data set) is determined from all isotherms.

- The monolayer induced electrolyte adsorption $\Delta\mathit{\Gamma}_{\mathrm{el}}$ for the medium concentration is calculated as

$$\Delta\mathit{\Gamma}_{\mathrm{el}} = -\rho_{\mathrm{w}} C_{\mathrm{el,m}} \mathit{\Gamma}_{\mathrm{s}} \left(\frac{\partial \Delta\mu_{\mathrm{s}}}{\partial p_{\mathrm{osm}}} \right)_{\pi} \tag{D.2}$$

  using the three-point finite central difference approximation. To avoid extrapolation beyond the experimental data set and differentiation within the phase transition region only the range ($\pi_{\mathrm{sp,min}}$,$\pi_{\mathrm{pt,max}}$) and ($\pi_{\mathrm{pt,min}}$,$\pi_{\mathrm{min,max}}$) is used.

- Alternatively, the monolayer induced electrolyte adsorption $\Delta\mathit{\Gamma}_{\mathrm{el}}$ for the medium concentration is calculated as

$$\Delta\mathit{\Gamma}_{\mathrm{el}} = \rho_{\mathrm{w}} C_{\mathrm{el,m}} \left(\frac{\partial \pi}{\partial p_{\mathrm{osm}}} \right)_{\mu_{\mathrm{s}}} \tag{D.3}$$

  using the three-point finite central difference approximation. To avoid extrapolation beyond the experimental data set and differentiation within the phase transition region only the range (0,$\Delta\mu_{\mathrm{pt,max}}$) and ($\Delta\mu_{\mathrm{pt,min}}$,$\Delta\mu_{\mathrm{min,max}}$) is used.

- The monolayer induced electrolyte adsorption at spreading is calculated as

$$\Delta\mathit{\Gamma}_{\mathrm{el,sp}} = \rho_{\mathrm{w}} C_{\mathrm{el,m}} \left(\frac{\partial \pi_{\mathrm{sp}}}{\partial p_{\mathrm{osm}}} \right)_{\mu_{\mathrm{s,sp}}} \tag{D.4}$$

  using the three-point finite central difference approximation.

E Types of interaction

Osmotic attraction. The monolayer-induced electrolyte adsorption $\Delta\mathit{\Gamma}_{\mathrm{el}}$ is the increase of the electrolyte excess with respect to the water's equimolecular plane when a monolayer is spread on the surface. The addition of a surfactant "expels" water from the surface. As a result the

equimolecular plane of water shifts towards the bulk. This results in a large non-specific osmotic contribution to $\Delta\Gamma_{\mathrm{el}}$.[4] In order to evaluate the specific interactions between the ions and lipids, one must first subtract the osmotic contribution. The plane of dielectric discontinuity is a natural choice of origin for ion adsorption models, since the electrostatic interactions (image force and in part the hydration force[5,6]) are invariant with respect to the it. Fortunately, the ε plane of discontinuity is approximately surfactant-independent.[4] This means that the monolayer-induced adsorption $\Delta\Gamma_{\mathrm{el}}^{\varepsilon}$ with respect to the plane of ε discontinuity is independent of the equimolecular shift and can be used to determine the presence of specific interactions. The experimentally accessible $\Delta\Gamma_{\mathrm{el}}$ is related to $\Delta\Gamma_{\mathrm{el}}^{\varepsilon}$ as[4]

$$\Delta\Gamma_{\mathrm{el}} = \frac{\Delta\Gamma_{\mathrm{el}}^{\varepsilon}}{1 - C_{\mathrm{el}}V_{\mathrm{el}}} + \frac{C_{\mathrm{el}}V_{\mathrm{s}}\Gamma_{\mathrm{s}}}{1 - C_{\mathrm{el}}V_{\mathrm{el}}}, \tag{E.1}$$

where V_{s} is the partial molar volume of the surfactant (headgroup) in a dense monolayer. Here, the bracket $(1 - C_{\mathrm{el}}V_{\mathrm{el}})$ stands for the effect of the electrolyte on the position of the equimolecular surface (for the studied system, a relatively small ion-specific effect), and $C_{\mathrm{el}}V_{\mathrm{s}}\Gamma_{\mathrm{s}}$ stands for the respective effect of the surfactant head groups. Eq. E.1 separates the adsorption into an ion-specific (determined by ion interactions) contribution $\Delta\Gamma_{\mathrm{el}}^{\varepsilon}$ and a non-specific $C_{\mathrm{el}}V_{\mathrm{s}}\Gamma_{\mathrm{s}}$. Except for strongly adsorbing ions, the non-specific osmotic effect tends to dominate.

The molar volume of the surfactant headgroup V_{s} is not a directly accessible parameter. The problem lies in the ambiguity of separation of the surfactant into a head and tail parts. Within our framework, the head part is the part of the molecule immersed below the plane of ε discontinuity. Similarly to our previous work, we estimate an upper and lower bound of V_{s}. Often, only the hydrocarbon part of the surfactant molecule is assumed to be the tail, while all functionalized segments of the molecule are assumed to be polar, i.e. part of the head. That is a reasonable upper bound for V_{s}. Assuming that everything up to the carbonyls of the acyl chains are immersed in the aqueous phase, we can take the average crystallographic volume 226 mL/mol[7] as an upper bound. The partial molar volume of the highly polar and zwitterionic phosphatidylcholine in water can be expected to be lower than the crystallographic volume. Therefore, our upper bound is probably slightly overestimated. As a lower bound we can assume that only the zwitterionic part

of the lipid is immersed in the aqueous phase, while the polar diglyceride is above the plane of ε discontinuity. Thus, we can take the crystallographic volume of phosphocholine, 122 mL/mol,[8] as a reasonable lower bound. That value is calculated from the crystallographic data of phosphocholine monohydrate[8] by subtracting the molar volume of water (18 mL/mol). This again ignores the possibility of negative interaction volume. Therefore, the lower bound is slightly underestimated.

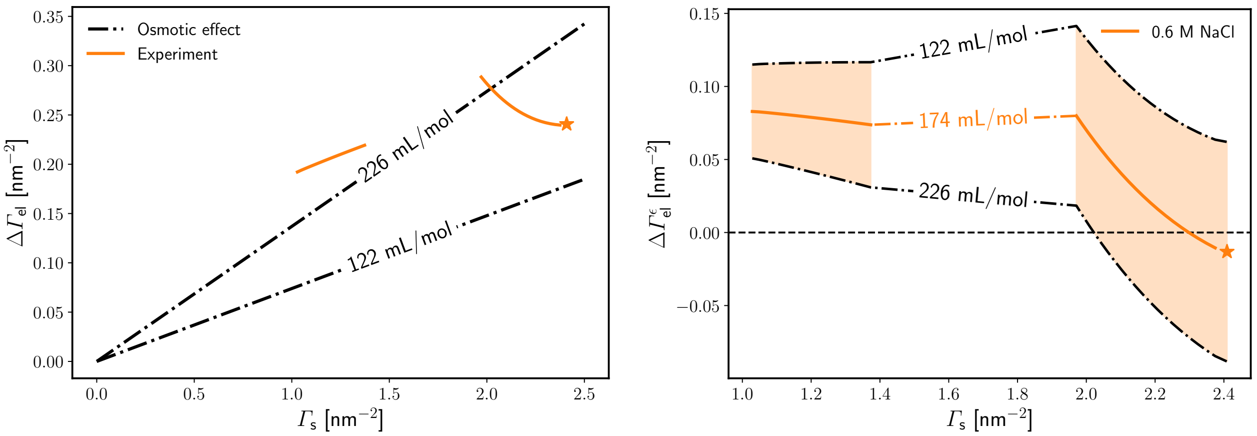

Figure E.1: (left) Monolayer-induced electrolyte adsorption $\Delta\Gamma_{\mathrm{el}}$ as a function of the adsorption Γ_{s} of DPPC at 0.6 M NaCl calculated at constant surfactant chemical potential μ_{s}. The star is calculated straight from the spreading pressure data. The dash-dotted lines are the osmotic contribution at the corresponding partial molar volumes V_{s} (see equation E.1). (right) Monolayer-induced adsorption of electrolyte with respect to the plane of dielectric discontinuity $\Delta\Gamma^{\varepsilon}_{\mathrm{el}}$ as a function of the adsorption of DPPC Γ_{s} at 0.6 M NaCl. $\Delta\Gamma^{\varepsilon}_{\mathrm{el}}$ is calculated at constant surface pressure. The partial molar volumes are indicated on each line. The semi-transparent areas highlight the implicit uncertainty coming from V_{s}.

This allows us to put bounds on the osmotic term in eq. E.1. Figure E.1 (left) compares the linear osmotic effect ($C_{\mathrm{el}}V_{\mathrm{s}}\Gamma_{\mathrm{s}}$) with the adsorption $\Delta\Gamma_{\mathrm{el}}$ obtained from the experimental data. As it can be seen, the shift of the equimolecular plane of water accounts for at least half of the monolayer-induced adsorption, i.e. it is the dominant effect behind the $\Delta\Gamma_{\mathrm{el}}$ vs Γ_{s} isotherm. Eq. E.1 and the bounds of V_{s} can be used to evaluate the ion-specific contribution to $\Delta\Gamma_{\mathrm{el}}$: the monolayer-induced electrolyte adsorption with respect to the dielectric plane of ε discontinuity, $\Delta\Gamma^{\varepsilon}_{\mathrm{el}}$. The results are presented in figure E.1 (right). Unlike $\Delta\Gamma_{\mathrm{el}}$, the sign of $\Delta\Gamma^{\varepsilon}_{\mathrm{el}}$ directly corresponds to repulsion ($\Delta\Gamma^{\varepsilon}_{\mathrm{el}} < 0$) or attraction ($\Delta\Gamma^{\varepsilon}_{\mathrm{el}} > 0$) between the ions and the lipids. As it can be seen, for most cases in the current system $\Delta\Gamma^{\varepsilon}_{\mathrm{el}}$ is positive, i.e. there is specific attraction between the NaCl and the DPPC.

Complexation. Often, the interaction between ions and lipids is discussed in terms of binding constants evoking the concept of complexation.[9] In such a case, the DPPC molecules may be treated as binding sites on the surface. Therefore, $\Delta\Gamma_{\mathrm{el}}^{\varepsilon}$ should increase when compressing the monolayer. In the simplest approximation, the increase is simply $\Delta\Gamma_{\mathrm{el}}^{\varepsilon} = KC_{\mathrm{el}}\Gamma_{\mathrm{s}}$, where K is an electrolyte-lipid association constant. However, even at the lower bound of V_{s} the slope $\partial\Delta\Gamma_{\mathrm{el}}^{\varepsilon}/\partial\Gamma_{\mathrm{s}}$ is either horizontal or negative (the lines connecting the semi-transparent areas in fig. E.1 are to guide the eye only; there is no experimental information between them). Thus, if there is a single-site complexation interaction, it seems to be counteracted by a repulsive interaction between the ions and the lipids.

Excluded volume. The volume occupied by the lipid head groups is not accessible to either water or the ions. The expulsion of water from the surface leads to the osmotic effect $C_{\mathrm{el}}V_{\mathrm{s}}\Gamma_{\mathrm{s}}$ discussed above, and electrolyte adsorption. On the other hand, the expulsion of ions leads to a drop in the ion depth-concentration profile and, thus, desorption. As a first approximation, we can assume that the surface ion concentration is proportional to the fraction of head group-free volume $1-\theta_{\mathrm{v}}$, where θ_{v} is the fraction of volume in the surface layer occupied by lipid head groups. θ_{v} can be found as a ratio of the monolayer density Γ_{s} and the maximum monolayer density (spreading pressure density $\Gamma_{\mathrm{s,sp}}$). However, even in the densest monolayer, there is some space available for the ions. Therefore, we will introduce a rough correction factor 0.91 (corresponding to discs in hexagonal lattice); $\theta_{\mathrm{v}} = 0.91\Gamma_{\mathrm{s}}/\Gamma_{\mathrm{s,sp}}$.

The headgroups of the monolayer can be assumed to form a layer of thickness $V_{\mathrm{s}}\Gamma_{\mathrm{s,sp}}$. On water|air, the surface concentration of electrolyte in this topmost layer is approximately $V_{\mathrm{s}}\Gamma_{\mathrm{s,sp}}C_{\mathrm{el}}$ plus the (negative) surface excess of electrolyte $\Gamma_{\mathrm{el}}^{\varepsilon,\mathrm{air}}$ (-0.11 nm^{-2} for 0.6 M NaCl[5]). Therefore, the excluded volume effect of the headgroups results in a drop of the surface electrolyte concentration by $(V_{\mathrm{s}}\Gamma_{\mathrm{s,sp}}C_{\mathrm{el}} + \Gamma_{\mathrm{el}}^{\varepsilon,\mathrm{air}}) \times 0.91\Gamma_{\mathrm{s}}/\Gamma_{\mathrm{s,sp}}$. The excluded volume diminishes also the number of ions associated with the lipid. The contribution $KC_{\mathrm{el}}\Gamma_{\mathrm{s}}$, from the ion-lipid association, to $\Delta\Gamma_{\mathrm{el}}$ is reduced to $KC_{\mathrm{el}}\Gamma_{\mathrm{s}}(1-0.91\Gamma_{\mathrm{s}}/\Gamma_{\mathrm{s,sp}})$. Synthesising the two effects, we obtain the following simple

adsorption isotherm:

$$\Delta\Gamma_{\rm el}^{\varepsilon} = KC_{\rm el}\Gamma_{\rm s}(1 - 0.91\Gamma_{\rm s}/\Gamma_{\rm s,sp}) - 0.91(V_{\rm s}\Gamma_{\rm s,sp}C_{\rm el} + \Gamma_{\rm el}^{\varepsilon,{\rm air}})\Gamma_{\rm s}/\Gamma_{\rm s,sp}. \quad \text{(E.2)}$$

The experimental data are in reasonable agreement with this formula. Figure E.2 (right) compares the experimentally derived $\Delta\Gamma_{\rm el}^{\varepsilon}$ at $V_{\rm s} = 226$ mL/mol with the predictions of the above simple model with $KC_{\rm el} = 0.25$. As it can be seen, the model cannot reproduce the shape of the isotherm with fine detail. However, it reproduces the overall trend surprisingly well. Furthermore, the value $KC_{\rm el} = 0.25$ implies that there is one ion couple adsorbed to every four lipid molecules (in the absence of osmotic effects). This is a reasonable physically meaningful result, thus, validating the model. Figure E.2 (left) also compares the experimental $\Delta\Gamma_{\rm el}$ with the theoretical one calculated by combining eqs. E.2 and E.1 ($V_{\rm s} = 226$ mL/mol and $KC_{\rm el} = 0.25$). Again the coincidence between the model and the experiment is quite good.

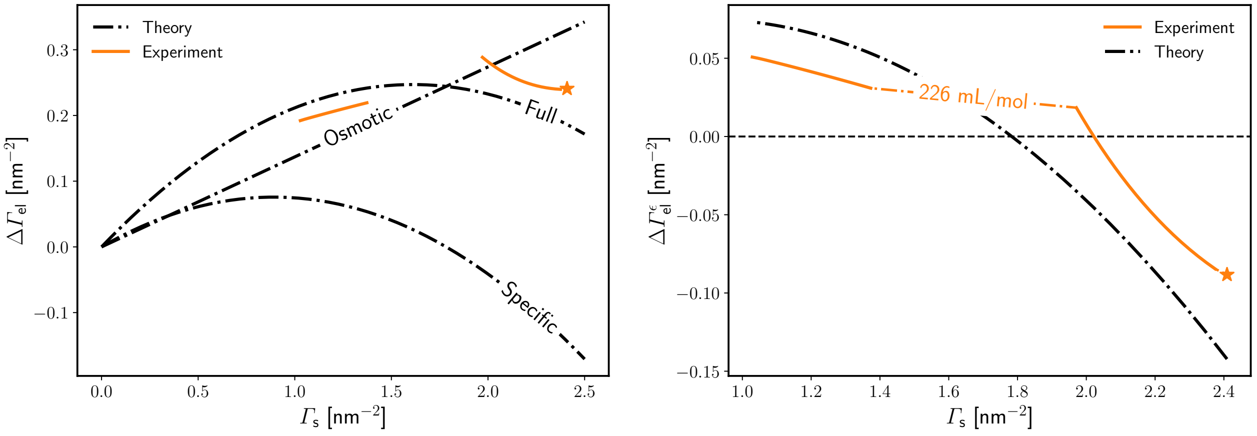

Figure E.2: (left) Monolayer-induced electrolyte adsorption $\Delta\Gamma_{\rm el}$ as a function of the adsorption $\Gamma_{\rm s}$ of DPPC at 0.6 M NaCl calculated at constant surfactant chemical potential $\mu_{\rm s}$. The star is calculated straight from the spreading pressure data. The dash-dotted lines are the theoretical model (eqs. E.1 and E.2) decomposed into a non-specific osmotic contribution, ion-specific contribution (eq. E.2) and the sum of the two; $V_{\rm s} = 226$ mL/mol, $KC_{\rm el} = 0.25$. (right) Monolayer-induced adsorption of electrolyte with respect to the plane of dielectric discontinuity $\Delta\Gamma_{\rm el}^{\varepsilon}$ as a function of the adsorption of DPPC $\Gamma_{\rm s}$ at 0.6 M NaCl calculated at constant surface pressure with $V_{\rm s} = 226$ mL/mol. The dashdotted line is the theoretical model (eq. E.2); $V_{\rm s} = 226$ mL/mol, $KC_{\rm el} = 0.25$.

References

(1) Adams, E. M.; Casper, C. B.; Allen, H. C. Effect of cation enrichment on dipalmitoylphosphatidylcholine (DPPC) monolayers at the air-water interface. *J. Colloid Interface Sci.* **2016**, *478*, 353–364.

(2) Peshkova, T. V.; Minkov, I. L.; Tsekov, R.; Slavchov, R. I. Adsorption of ions at uncharged insoluble monolayers. *Langmuir* **2016**, *32*, 8858–8871.

(3) Minkov, I. L.; Arabadzhieva, D.; Salama, I. E.; Mileva, E.; Slavchov, R. I. Barrier kinetics of adsorption–desorption of alcohol monolayers on water under constant surface tension. *Soft matter* **2019**, *15*, 1730–1746.

(4) Peychev, B.; Slavchov, R. I. Interactions between small inorganic ions and uncharged monolayers on the water/air interface. *J. Phys. Chem. B* **2023**,

(5) Slavchov, R. I.; Novev, J. K. Surface tension of concentrated electrolyte solutions. *J. Colloid Interface Sci.* **2012**, *387*, 234–243.

(6) Slavchov, R. I.; Novev, J. K.; Peshkova, T. V.; Grozev, N. A. Surface tension and surface $\Delta\chi$-potential of concentrated Z+: Z- electrolyte solutions. *J. Colloid Interface Sci.* **2013**, *403*, 113–126.

(7) Marsh, D. Molecular volumes of phospholipids and glycolipids in membranes. *Chem. Phys. Lipids* **2010**, *163*, 667–677.

(8) Nikawa, Y.; Fujita, K.; Noguchi, K.; Ohno, H. 2-(Trimethylazaniumyl) ethyl hydrogen phosphate (phosphocholine) monohydrate. *Acta Crystallogr. E* **2014**, *70*, o549–o549.

(9) Giner Casares, J. J.; Camacho, L.; Martín-Romero, M. T.; López Cascales, J. J. Effect of Na^+ and ${Ca_2}^+$ ions on a lipid Langmuir monolayer: an atomistic description by molecular dynamics simulations. *ChemPhysChem* **2008**, *9*, 2538–2543.
